# Difficulties in emotional regulation in a Tunisian university setting

**DOI:** 10.1192/j.eurpsy.2024.1186

**Published:** 2024-08-27

**Authors:** S. Boudriga, M. Lagha, M. Methni, Y. Ben youssef, I. Ben romdhane, W. Homri, R. Labbane

**Affiliations:** ^1^Department C; ^2^Psychiatry C, Razi Hospital, Manouba, Tunisia

## Abstract

**Introduction:**

Emotion regulation is the conscious or automatic control of emotions to adapt, cope, and maintain well-being. Effective emotion regulation is central to mental health, impacting work, and relationships. University students, facing academic pressures and social transitions, represent a unique demographic where emotion regulation challenges are particularly relevant.

**Objectives:**

This study aimed to explore the emotion regulation difficulties in university students.

**Methods:**

A descriptive study was led from August to September 2023. An online questionnaire was distributed to a population of Tunisian university students. We administered a socio-demographic questionnaire and the Arabic version of the difficulties in emotion regulation scale short form (DRES-SF), a self-report measure developed to assess clinically relevant difficulties in emotion regulation.

**Results:**

Participants in this study consisted of 307 undergraduate students, with 78.1% being women and 21.9% men, representing various academic disciplines at Tunis el Manar University in Tunisia. The mean age of the participants was 22 years, with a standard deviation of 2.84.In the assessment of emotional regulation difficulties, participants reported a mean total score of 42.47 ± 12.68. Participants who repeated years in college had more difficulties in emotional regulation (p<0.05). Limited access to emotion regulation strategies had a mean score of 7.64 ± 3.0, while nonacceptance of emotional responses was rated at 7.40 ± 3.17. Additionally, impulse control difficulties were reported with a mean score of 6.46 ± 3.31, and difficulties in engaging in goal-directed behavior were observed with a mean score of 9.44 ± 3.18. Moreover, participants expressed a lack of emotional awareness, which was quantified with a mean score of 8.45 ± 2.69, and a lack of emotional clarity, which yielded a mean score of 7.12 ± 2.69. Additionally, a significant association was noted between gender (p < 0.05), age (p < 0.05), and the lack of emotional awareness, suggesting potential gender and age-related variations in emotional regulation difficulties within this university sample.

**Conclusions:**

Overall, these findings suggest the necessity of emotion regulation training in the university setting. Further studies are important to understand the impact of emotional regulation difficulties.

**Disclosure of Interest:**

None Declared

